# The Role of Maternal Stress in Early Pregnancy in the Aetiology of Gastroschisis: An Incident Case Control Study 

**DOI:** 10.1371/journal.pone.0080103

**Published:** 2013-11-08

**Authors:** Stephen R. Palmer, Annette Evans, Hannah Broughton, Simon Huddart, Mark Drayton, Judith Rankin, Elizabeth S. Draper, Alan Cameron, Shantini Paranjothy

**Affiliations:** 1 Cochrane Institute of Primary Care and Public Health, School of Medicine, Cardiff University, Cardiff, United Kingdom; 2 Cardiff and Vale University Health Board, Cardiff, United Kingdom; 3 Institute of Health and Society, Newcastle University, Newcastle, United Kingdom; 4 Department of Health Sciences, University of Leicester, Leicester, United Kingdom; 5 The University of Glasgow, Glasgow, United Kingdom; 6 The Ian Donald Fetal Medicine Unit, Southern General Hospital, Glasgow, United Kingdom; UCL Institute of Child Health, University College London, United Kingdom

## Abstract

**Objective:**

The incidence of gastroschisis, a congenital anomaly where the infant abdominal wall is defective and intestines protrude from the abdominal cavity, is increasing in many countries. The role of maternal stress in some adverse birth outcomes is now well established. We tested the hypothesis that major stressful life events in the first trimester are risk factors for gastroschisis, and social support protective, in a case-control study in the United Kingdom.

**Methods:**

Gastroschisis cases and three controls per case (matched for maternal age) were identified at routine 18-20 week fetal anomaly ultrasound scan, in 2007-2010. Face to face questionnaire interviews were carried out during the antenatal period (median 24 weeks gestation) asking about serious stressful events and social support in the first trimester. Data were analysed using conditional logistic regression.

**Results:**

Two or more stressful life events in the first trimester (adjusted OR 4.9; 95% CI 1.2-19.4), and moving address in the first trimester (aOR 4.9; 95% CI 1.7-13.9) were strongly associated with risk of gastroschisis, independent of behavioural risk factors including smoking, alcohol, and poor diet. Perceived availability of social support was not associated with reduced risk of gastroschisis (aOR 0.8; 95% CI 0.2-3.1).

**Conclusions:**

Stressful maternal life events in the first trimester of pregnancy including change of address were strongly associated with a substantial increase in the risk of gastroschisis, independent of stress related high risk behaviours such as smoking, alcohol consumption and poor diet. This suggests that stress pathways are involved in the aetiology of gastroschisis.

## Introduction

**Figure 1:**
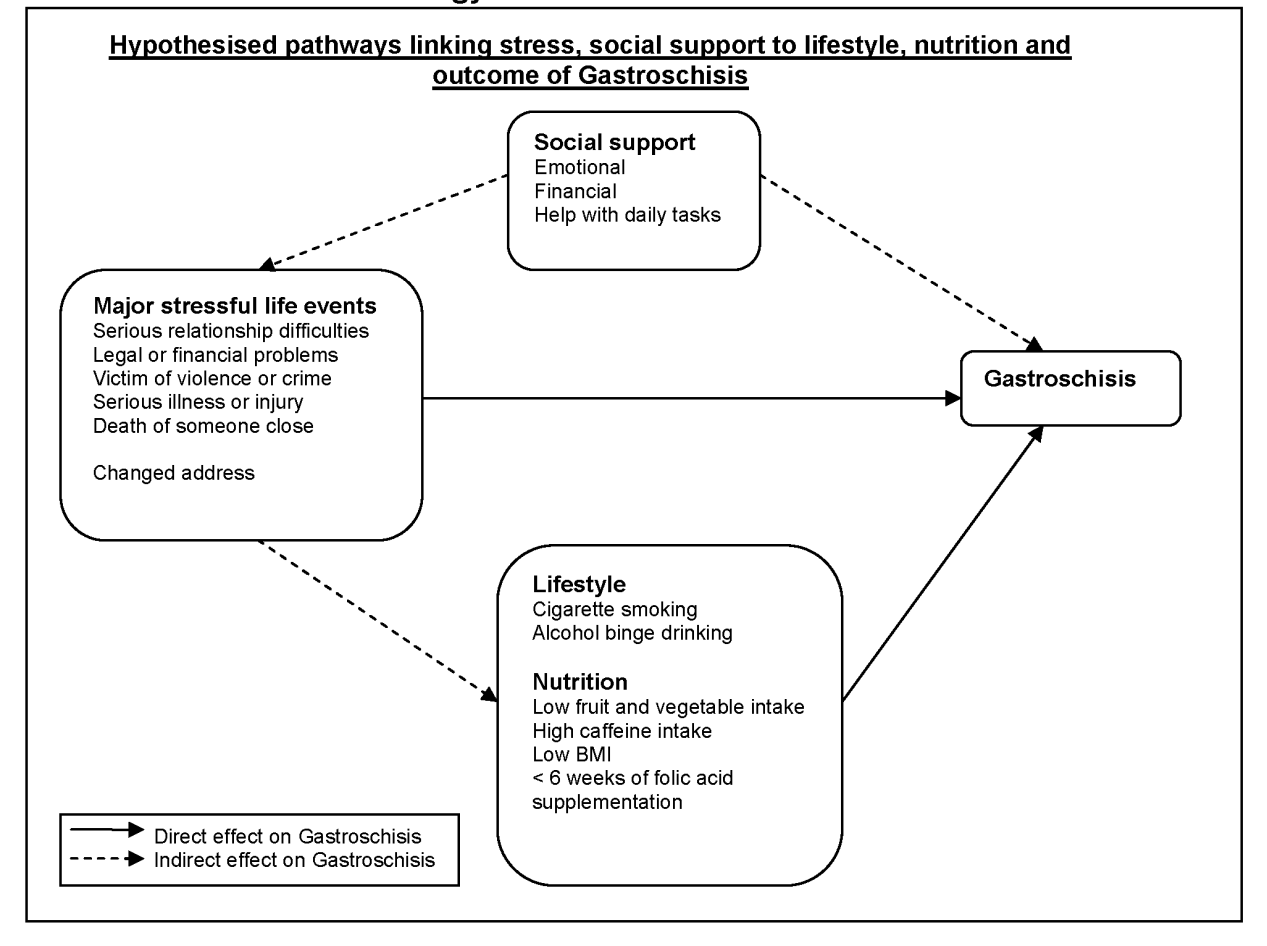
Hypothesised pathway diagram for Stress, Social support, Lifestyle and Nutrition in the aetiology of Gastroschisis.

The aetiology of gastroschisis, a congenital anomaly of the abdominal wall with herniation of the intestines and serious morbidity [1,2], is unknown [3]. Birth prevalence is reported to have increased in many countries over the last 15 years [4,5]. Evidence of a degree of clustering of cases [6] which can lead to public alarm [7] raised the question of possible point sources of pollution as a causative factor, but to date no definitive environmental exposures have been identified. Case control studies of gastroschisis have consistently found associations with the behaviour-related risk factors of low maternal BMI, younger age of mother, smoking [8], and more recently risks associated with recreational drug use [9], genitourinary infection [10] and diet poor in fruit and vegetables, and reduced folic acid intake [11] have been reported. In addition to exploring these risk factors we have drawn on growing scientific interest in the potential role of maternal stress in causing adverse birth outcomes [12,13] including congenital anomalies [14-16]. In this analysis we tested the hypothesis that major stressful life events in the first trimester were associated with increased risk of gastroschisis, and that social support was protective (Figure 1), using data from an incident case control study [11]. 

## Method

The case control study was carried out in five regions of the United Kingdom [11]. A full description of the methods has been reported previously [11]. Briefly, women were recruited between July 2007 and February 2010. Cases were identified following the ultrasound scan offered routinely to all pregnant women in England and Wales at 18-20 weeks gestation and intended to identify fetal anomalies. We included singleton pregnancies in which uncomplicated gastroschisis (ICD-10 Q79.3) was diagnosed. For women in Scotland routine ultrasound scan at 18-20 weeks was not offered routinely in all areas so instead we followed up mothers whose routine serum alpha fetoprotein level was high and who then went on to have ultrasound investigations. We excluded babies with multiple anomalies. We recruited three controls for each case using routine National Health Service records of the antenatal clinics where the case mothers attended. We matched for maternal age within 1 year (or within 3 years where this was not obtainable). Ethical approval was given by the Research Ethics Committee for Wales (05/MRE09/97) and required informed written consent was obtained from all the women. 

Women were interviewed by a trained interviewer using a standard structured questionnaire. We sought to minimise recall bias by asking mothers to use diaries and calendars to think about the period in question. Questions on stressful maternal events were taken from The U.S. National Birth Defects Prevention Study [16] which were based on several validated assessment tools for stressful life events [14,16-18] and social support [19,20]. Questions covered five stressful life events during the first trimester: serious relationship difficulties with husband or partner (including separation or divorce), the woman or partner having serious financial or legal problems, the woman or someone close was a victim of violence or violent crime, the woman or someone close having a serious illness or injury, and someone close died. The definition of “someone close” was decided by the participant. We also included as a stressful event change of address in the first trimester [21]. Questions about social support covered availability of emotional support with problems and decisions, financial help (with bills, food and clothes), help with daily tasks (such as grocery shopping, child care and cooking). Other questions included socio-demographic characteristics, change of partner between pregnancies, planned pregnancy, housing, nutrition (including use of folic acid supplements), infections, medication and recreational drug use, social events such as parties, hobbies, patterns of caffeine intake (cups of tea, coffee, caffeinated drinks a day), alcohol intake and cigarette smoking, during the first twelve weeks of pregnancy. To assess socio-economic status we used the National Statistics Socio-Economic grouping (NS-SEC) [22].

## Statistical Analysis

In other studies of birth defects the cumulative number of stressful life events has been found to be associated with increased risk; therefore following examination of the distribution, we categorised the number of stressful events into 0, 1, 2+ events. We fitted univariate and multivariable conditional logistic regression models using STATA version 10 [23] (Statacorp, College Station, Texas). Most cases and controls reported having all three types of social support and therefore we categorised social support into a binary Yes (if they had all three types of social support) / No (if they did not have at least one type of social support) as the variable of interest in the logistic regression modelling. We found that units of alcohol consumed per week and reported binge drinking (greater or equal to 6 units in one sitting/day) were highly correlated, so we ran models using each alcohol variable in turn. Odds ratios were very similar and we report the data for the model using binge drinking. 

We included two-way interaction terms between the number of stressful life events and the behavioural risks of smoking, binge drinking and caffeine intake, fruit and vegetable intake and duration of folic acid supplementation in the first trimester, and tested if these terms improved the fit of the model to the data using the log likelihood ratio test [24]. 

## Results

Of the 124 eligible cases identified 91(73%) agreed to participate in the study as did 217 (70%) of 310 potential controls. The mean age was 23 years (SD 4.6 years) for cases and 23 years (SD 5.0 years) for controls. In univariate analysis, cases were almost twice as likely to report individually three of the five major stressful life events in the first trimester: serious relationship difficulties, legal or financial problems, or victim of violence or crime (Table 1). 

**Table 1:** Characteristics of Gastroschisis Cases and Controls (n=308).

**Characteristic**		**Participant Status**				**Univariate Odds Ratio (95% CI**)		
		**Control**		**Case**				
		**n (%)**		**n** (**%**)				
Stress 1: Serious relationship difficulties for you / partner; first trimester	No	171	(78.8)	56	(61.5)	1.0		
	Yes	46	(21.2)	35	(38.5)	2.2	(1.3,	3.9)
Stress 2: Legal or financial problems for you / partner; first trimester	No	188	(86.6)	70	(76.9)	1.0		
	Yes	29	(13.4)	21	(23.1)	2.0	(1.0,	3.9)
Stress 3: Victim of violence or crime for you / someone close; first trimester	No	204	(94.0)	72	(79.1)	1.0		
	Yes	13	(6.0)	19	(20.9)	4.3	(1.7,	10.9)
Stress 4: Serious illness or injury for you / someone close; first trimester	No	185	(85.3)	76	(83.5)	1.0		
	Yes	32	(14.7)	15	(16.5)	1.1	(0.5,	2.1)
Stress 5: Death of someone close; first trimester	No	202	(93.1)	81	(89.0)	1.0		
	Yes	15	(6.9)	10	(11.0)	1.8	(0.8,	4.3)
Number of stressful life events (from 5 listed above)	0	122	(56.2)	37	(40.7)	1.0		
	1	66	(30.4)	26	(28.6)	1.3	(0.7,	2.4)
	2+	29	(13.4)	28	(30.8)	2.8	(1.4,	5.5)
Social support 1: Anyone to give emotional support to you if needed at this time; first trimester	No	9	(4.1)	11	(12.1)	1.0		
	Yes	208	(95.9)	80	(87.9)	0.3	(0.1,	0.8)
Social support 2: Anyone to give financial support to you if needed at this time; first trimester	No	19	(8.8)	13	(14.3)	1.0		
	Yes	198	(91.2)	78	(85.7)	0.6	(0.3,	1.4)
Social support 3: Anyone to help with daily tasks for you if needed at this time; first trimester	No	9	(4.1)	8	(8.8)	1.0		
	Yes	208	(95.9)	83	(91.2)	0.5	(0.2,	1.4)
Social support available (as defined above)	No (lack of 1+ support)	28	(12.9)	21	(23.1)	1.0		
	Yes (all 3 supports)	189	(87.1)	70	(76.9)	0.5	(0.3,	1.0)
Same address for first 12 weeks of pregnancy	Yes	177	(81.6)	54	(59.3)	1.0		
	No	40	(18.4)	37	(40.7)	3.3	(1.8,	5.8)
Changed Partner during this pregnancy from previous pregnancies	No	80	(36.9)	29	(31.9)	1.0		
	Yes	22	(10.1)	21	(23.1)	2.7	(1.2,	6.0)
Lives with	First pregnancy	115	(53.0)	41	(45.1)	0.9	(0.5,	1.6)
	Partner and/or children	141	(65.0)	56	(61.5)	1.0		
	Parents and/or siblings	46	(21.2)	20	(22.0)	1.0	(0.5,	2.3)
	Live alone / Other	30	(13.8)	15	(16.5)	0.9	(0.4,	2.1)
Pregnancy desire	Did not want to be pregnant at all	19	(8.8)	8	(8.8)	1.4	(0.5,	3.6)
	Surprise	5	(2.3)	5	(5.5)	2.7	(0.7,	10.6)
	Wanted to become pregnant then	111	(51.2)	41	(45.1)	1.0		
	Wanted to wait till later	82	(37.8)	37	(40.7)	1.3	(0.7,	2.4)
Parity	None	139	(64.4)	58	(63.7)	1.0		
	≥ 1 child	77	(35.6)	33	(36.3)	1.2	(0.7,	2.1)

There were no significant differences between cases and controls in the frequency of injury, illness or death in someone close. Forty one percent of cases reported a change of address in the first trimester compared to 18% of controls (OR 3.3; 95%CI 1.8-5.8). Cases were also more likely than controls to have changed partners for the current pregnancy (OR 3.0; 95% CI 1.4-6.4). Controls were more likely than cases to report having someone to provide emotional support (OR 0.3; 95% CI 0.1-0.8) but both had similar levels of financial support and help with daily tasks. Stressful life events were not consistently related to other risk factors (Table 2, Table S1). 

**Table 2:** Lifestyle, socio-demographic and nutrition characteristics by summative stress and residential move (n=308).

		**Sum of major stressful life events:- Serious relationship difficulties, legal or financial problems, victim of abuse, serious illness or injury, death of someone close; in first trimester**													**Same address for first 12 weeks of pregnancy**							
		**0**				**1**				**2+ stresses**					**Yes**				**No**			
		**Control**		**Case**		**Control**		**Case**		**Control**		**Case**			**Control**		**Case**		**Control**		**Case**	
		**n**	**(%)**	**n**	**(%)**	**n**	**(%)**	**n**	**(%)**	**n**	**(%)**	**n**	**(%)**		**n**	**(%)**	**n**	**(%)**	**n**	**(%)**	**n**	**(%)**
Cigarettes smoked per day in first trimester	0	68	(55.7)	9	(24.3)	33	(50)	8	(30.8)	17	(58.6)	9	(32.1)		100	(56.5)	15	(27.8)	18	(45.0)	11	(29.7)
	0-10	38	(31.1)	20	(54.1)	23	(34.8)	8	(30.8)	10	(34.5)	14	(50.0)		52	(29.4)	28	(51.9)	19	(47.5)	14	(37.8)
	>10	16	(13.1)	8	(21.6)	10	(15.2)	10	(38.5)	2	(6.9)	5	(17.9)		25	(14.1)	11	(20.4)	3	(7.5)	12	(32.4)
Binge drinker (≥6 units in one sitting/day) in first trimester	No	72	(59.0)	17	(45.9)	42	(66.7)	12	(48.0)	20	(69.0)	11	(39.3)		113	(64.9)	24	(44.4)	21	(52.5)	16	(44.4)
	Yes	50	(41.0)	20	(54.1)	21	(33.3)	13	(52.0)	9	(31.0)	17	(60.7)		61	(35.1)	30	(55.6)	19	(47.5)	20	(55.6)
Maternal age at childbirth	< 20	35	(28.7)	12	(32.4)	15	(22.7)	7	(26.9)	5	(17.2)	7	(25.0)		44	(24.9)	18	(33.3)	11	(27.5)	8	(21.6)
	20 - 24.99	45	(36.9)	15	(40.5)	34	(51.5)	11	(42.3)	18	(62.1)	17	(60.7)		75	(42.4)	23	(42.6)	22	(55.0)	20	(54.1)
	25+	42	(34.4)	10	(27.0)	17	(25.8)	8	(30.8)	6	(20.7)	4	(14.3)		58	(32.8)	13	(24.1)	7	(17.5)	9	(24.3)
NS-SEC classification (of mother)	Managerial & professional / intermediate	34	(27.9)	7	(18.9)	17	(25.8)	3	(11.5)	7	(24.1)	2	(7.1)		47	(26.6)	7	(13.0)	11	(27.5)	5	(13.5)
	Routine & manual	36	(29.5)	17	(45.9)	22	(33.3)	12	(46.2)	8	(27.6)	11	(39.3)		57	(32.2)	24	(44.4)	9	(22.5)	16	(43.2)
	Student	10	(8.2)	3	(8.1)	4	(6.1)	2	(7.7)	3	(10.3)	1	(3.6)		15	(8.5)	4	(7.4)	2	(5.0)	2	(5.4)
	Unemployed	42	(34.4)	10	(27)	23	(34.8)	9	(34.6)	11	(37.9)	14	(50.0)		58	(32.8)	19	(35.2)	18	(45.0)	14	(37.8)
Typical number of fruit or vegetables portions eaten per week (excluding potatoes)	0 - 6 portions	28	(23.1)	13	(35.1)	16	(24.2)	9	(37.5)	3	(10.7)	9	(33.3)		37	(21.1)	20	(37.7)	10	(25.0)	11	(31.4)
	7 - 13 portions	30	(24.8)	11	(29.7)	18	(27.3)	9	(37.5)	7	(25.0)	7	(25.9)		43	(24.6)	17	(32.1)	12	(30.0)	10	(28.6)
	14 - 20 portions	24	(19.8)	8	(21.6)	15	(22.7)	1	(4.2)	9	(32.1)	5	(18.5)		41	(23.4)	8	(15.1)	7	(17.5)	6	(17.1)
	21+ portions	39	(32.2)	5	(13.5)	17	(25.8)	5	(20.8)	9	(32.1)	6	(22.2)		54	(30.9)	8	(15.1)	11	(27.5)	8	(22.9)
Duration in weeks of folic acid supplementation during first trimester	< 6 out of first 12 weeks	36	(29.5)	17	(45.9)	20	(30.3)	11	(42.3)	7	(24.1)	18	(64.3)		46	(26.0)	30	(55.6)	17	(42.5)	16	(43.2)
	≥ 6 out of first 12 weeks	86	(70.5)	20	(54.1)	46	(69.7)	15	(57.7)	22	(75.9)	10	(35.7)		131	(74.0)	24	(44.4)	23	(57.5)	21	(56.8)
Social support available (Emotional, Financial, Help with daily tasks)	No (lack of 1+ support)	14	(11.5)	4	(10.8)	8	(12.1)	6	(23.1)	6	(20.7)	11	(39.3)		20	(11.3)	12	(22.2)	8	(20.0)	9	(24.3)
	Yes (all 3 supports)	108	(88.5)	33	(89.2)	58	(87.9)	20	(76.9)	23	(79.3)	17	(60.7)		157	(88.7)	42	(77.8)	32	(80.0)	28	(75.7)

In multivariable analysis having two or more major stressful life events as well as changing address in the first trimester were both strongly associated with increased odds of gastroschisis, independent of each other and of behavioural risk factors (Table 3). Odds ratios were similar in magnitude to the independent association with cigarette smoking. Social support was not independently associated with reduced risk of gastroschisis after adjustment for stressful life events. The risks associated with BMI, binge drinking, consumption of fruit or vegetables and folic acid supplements were unchanged from the estimates modelled without the stress variables. Socio-economic group, caffeine intake and change of partner were not associated with increased risk in the multivariate models. 

**Table 3:** Adjusted conditional odds ratios for the association between stress, socio-demographics, lifestyle, nutritional factors and gastroschisis (complete case).

**Characteristic**		**Univariate Odds Ratio (95% CI**)			**Adjusted** ^a^ **Odds Ratio** (**95% CI**)		
Number of major stressful life events:- Serious relationship difficulties / Legal or financial problems / Victim of violence / Injury or illness / Death of someone close; first trimester	0	1.0			1.0		
	1	1.3	(0.7,	2.4)	1.0	(0.4,	2.8)
	2+	2.8	(1.4,	5.5)	4.9	(1.2,	19.4)
Social support available:- Emotional, financial, Help with daily tasks; first trimester	No (lack of 1+ support)	1.0			1.0		
	Yes (all 3 supports)	0.5	(0.3,	1.0)	0.8	(0.2,	3.1)
Same address for first 12 weeks of pregnancy	Yes	1.0			1.0		
	No	3.3	(1.8,	5.8)	4.9	(1.7,	13.9)
Changed partner during this pregnancy from previous pregnancies	No	1.0			1.0		
	Yes	2.7	(1.2,	6.0)	3.5	(0.8,	16.0)
	First pregnancy	0.9	(0.5,	1.6)	1.8	(0.6,	5.5)
NS-SEC classification (of mother)	Managerial and professional / intermediate	1.0			1.0		
	Routine and manual occupations	3.2	(1.4,	7.3)	3.6	(0.9,	15.5)
	Unemployed	2.6	(1.1,	6.2)	1.2	(0.3,	5.1)
	Student	1.9	(0.5,	6.7)	1.0	(0.8,	6.0)
Cigarettes smoked per day in first trimester	0	1.0			1.0		
	> 0 - 10	2.7	(1.5,	5.0)	3.9	(1.3,	11.4)
	> 10 cigarettes	3.5	(1.6,	7.6)	4.3	(1.0,	18.0)
Woman binge drinker (≥ 6 units^b^ in one sitting/day) in first trimester	No	1.0			1.0		
	Yes	2.0	(1.2,	3.4)	1.6	(0.6,	4.2)
Body Mass index	Underweight (<18.5)	1.8	(0.7,	4.7)	3.4	(0.6,	20.8)
	Normal (18.5-24.9)	1.0			1.0		
	Overweight (25-29.9)	0.8	(0.4,	1.6)	0.8	(0.3,	2.5)
	Obese(30+)	0.3	(0.1,	0.8)	0.1	(0.03,	0.7)
Typical number of fruit or vegetables portions eaten per week (excluding potatoes) during the first trimester	≤ 6 portions a week	1.0			1.0		
	7-13	0.7	(0.3,	1.6)	0.4	(0.1,	1.5)
	14-20	0.3	(0.2,	0.8)	0.2	(0.06,	0.9)
	21+	0.2	(0.1,	0.5)	0.2	(0.04,	0.8)
Duration in weeks of folic acid supplementation during first trimester	< 6 out of first 12 weeks	1.0			1.0		
	≥ 6 out of first 12 weeks	0.3	(0.2,	0.6)	0.3	(0.1,	0.8)
Minimum caffeine intake per week in first trimester	0 - 1400mg	1.0			1.0		
	> 1400 - 2800mg	1.4	(0.8,	2.6)	0.7	(0.3,	1.8)
	> 2800 - 4200mg	1.1	(0.4,	2.6)	0.3	(0.1,	1.5)
	>4200mg	3.3	(1.5,	7.4)	0.9	(0.2,	3.8)
Nausea or vomiting during the first trimester	No	1.0			1.0		
	Yes	0.4	(0.2,	0.9)	0.4	(0.1,	1.5)

a Adjusted for all variables in shown in the table using conditional logistic regression

b A UK standard unit of alcohol is 10ml or 8g of pure alcohol. This is equivalent to half a pint of beer, one sixth of a gill of spirits, or a glass of wine (125ml). We have included this information in the methods section.

There was no evidence of significant two-way interactions between the stress variables and the behavioural risk factors of cigarette smoking, binge drinking, caffeine intake, fruit or vegetable intake or folic acid use. In order to account for potential residual confounding with maternal age we repeated the analysis including maternal age as a continuous variable but this did not substantially alter the results.

## Discussion

The embryological pathogenesis of gastroschisis is uncertain [1,25-28]. Hypotheses have mainly focussed on vascular abnormality and consequent infarction and necrosis of the body wall at about 6-10 weeks of gestation, but Feldkamp et al [26] have argued that animal model data suggest that the causes may operate even earlier in embryogenesis than previously thought and propose that gastroschisis is caused by abnormal folding of the body wall resulting in a ventral body wall defect and gut herniation at 3-5 weeks post conception. Our study was aimed at pin pointing time specific aetiological exposures although the closest we could come realistically in interview was to specify the first trimester period. 

Epidemiological studies have identified several potential risk factors but only a few have been found consistently and these are low maternal age, low BMI and smoking [8]. Associations with alcohol intake is less consistent [8,11]. The association with low BMI may suggest nutritional deficiency, and recently we have reported an association with low intake of fruit and vegetables and reduced duration of folic acid supplementation in the first trimester which were as strongly linked to gastroschisis as smoking and low maternal age [11]. However, the association with nutritional factors was independent of maternal BMI. Indeed our data suggested that rather than low BMI being a risk factor, obesity was protective. The consistent and strong independent association with younger maternal age as well as the increasing incidence in gastroschisis in recent years in many countries suggests a role for other modern life style related risk factors. We considered the potential role of recreational and across the counter drugs, as have others [9,29,30] and also the relatively new phenomenon of binge drinking in women, but these were not independently associated with gastroschisis [11]. 

We hypothesised that stressful maternal life events increase the risk of gastroschisis, possibly through biochemical and immunological stress pathways. Similar hypotheses have been proposed for adverse perinatal events such as low birthweight and prematurity [12,13,31,32] and some other congenital anomalies [14], particularly cleft palate and cleft lip [15,16]. There are a number of ways in which maternal stress during pregnancy may lead to gastroschisis. Stress may lead to increase in behaviours already implicated as risk factors such as cigarette smoking or poor nutrition [16]. We were able to take account of these factors in our analysis and maternal stress had a strong association with gastroschisis, independent of the risks associated with cigarette smoking or poor nutrition. Increased levels of cortisol due to stress may have a direct effect on the fetus, but to our knowledge there is no evidence for this from animal models. Cortisol plays a role in vascular and thrombotic pathways, both of which have been hypothesised as key factors in the development of gastroschisis [25,27]. More recently, high levels of oestrogen have been proposed to play a role [33]. High levels of oestrogen are associated with increased risk of thrombosis, and oestrogen is also associated with negative reactions to stress [34], such that women who were already at increased risk of gastroschisis due to high level of oestrogen may have perceived more stress. Finally, cortisol is known to be associated with dysregulation of the immune and inflammatory pathway but its effect in pregnancy is less well known [35]. It is possible that these immune and inflammatory pathways play a role in the development of gastroschisis [8]. 

As far as we are aware this is the first study to test this hypothesis in gastroschisis. We found univariate associations between gastroschisis and the individual life events of serious relationship difficulties, legal or financial problems for the women or their partners, and with the women or someone close being a victim of violence or crime. Access to emotional support was associated with a reduced risk. However, in multivariate analysis we found that two or more major life events compared to one or less, and moving home in the first trimester, each were independent of each other and of maternal age, BMI, smoking and nutritional factors. The strength of the associations we found for gastroschisis was similar to the independent effects of smoking or poor diet and suggests a major role for direct stress pathways independent of other lifestyle factors. Using the estimates from the multivariate model, though with very wide confidence intervals, the risk to a woman who smokes and who has two or more stressful life events in the first trimester was 21.0 (95% CI 2.2-203.5). If in addition she moved address the risk estimate increases to 81.3 (95% CI 3.2-2083.2). Although social support has been proposed as a moderating factor for stress we did not find that it was independently associated in any protective way. There was no evidence of interactions between these stress variables and other variables included in the model. 

### Strengths

Our study population was likely to be representative of all case and control pregnancies that reach 18 weeks of gestation in the UK since all women are offered antenatal USS in routine universal NHS antenatal care. Completeness of ascertainment was checked with congenital anomaly registers and fetal medicine units, and the response rates of case and control mothers was high. Most previous studies of risk factors that would by definition have to operate very early in pregnancy were based on interview data after birth and therefore involved a long period of recall, but we interviewed women on average at 24 weeks gestation so that the recall period was minimised. Differential bias in recall and rumination between cases and controls was minimised by using diaries and calendars and by ensuring that cases and controls were interviewed for similar duration at similar gestational ages [11]. The stress questions that were included in face to face interviews were standard questions from the USA birth defect study [16] allowing comparison with other similar studies. 

### Limitations

The major concern with the retrospective nature of the case control study method is reverse causality, the possibility that women who have been given a diagnosis of gastroschisis are more likely to recall or interpret life events in earlier pregnancy as stressful. However, we did not rely upon the women’s own judgement of what was a stressful event. Rather we sought to identify life events that have been well studied by others and were likely to be readily recalled and which could be recorded as Yes/No answers. The nature of these events was such that they were often still a problem at the time of interview and therefore recall was unlikely to be biased by knowledge of gastroschisis status. We believe also that the fact of a change of address in the first trimester was also unlikely to be subject to serious differential recall bias between cases and controls. However, we did not have any potential biochemical, endocrine or inflammatory markers of potential stress pathways. We were also unable to fully explore the role of genitourinary infections in this study. We did ask about infections during the first trimester, but the majority of these were upper respiratory tract infections. A very small number of cases and controls (<5) reported genitourinary infections, but these were not verified with microbiological reports. 

### Interpretation

Stressful life events may operate through two main pathways. Firstly stress may lead to unhealthy behaviours such as smoking, alcohol and poor diet [36,37] and secondly that maternal stress may operate through direct biochemical and immunological pathways to affect embryological development [32,38]. We did not find evidence of a strong correlation between cigarette smoking, alcohol and poor diet and higher levels of stress; however we adjusted for these behavioural risk factors in multivariate analysis, and maternal stress was nevertheless independently associated with gastroschisis. These risk factors are more common in more socio-economically disadvantaged women but adjusting for socio-economic group did not reduce odds ratios. 

Our data suggest that serious consideration should be given to direct stress pathways in the aetiology of gastroschisis as has been shown for premature birth [38] and impaired neurodevelopment of the child [39]. Hansen et al [14] studied all births in Denmark from 1980-1992 and described a 50% increased risk of cranial-neural-crest malformations but not other malformations in women whose partners or children had died or had been admitted to hospital with severe disease in the first trimester, adjusting for a range of maternal factors but not for maternal weight or diet. We asked mothers about deaths and severe illness in those close to them but the rarity of these events precluded us assessing risk. Carmichael et al [16] have since reported data from California on an increased risk of cleft lip, cleft palate, anenecephaly, spina bifida, and Tetralogy of Fallot (though they did not include gastroschisis) using an 18 point inventory of stressful life events, controlling for weight, smoking, alcohol and folic acid intake but not for fruit and vegetables in the diet. They found that an increase in stress score was associated with increased risk in a dose-response manner. 

Many of these stress factors are highly correlated so we included wider ranging questions to include all events in a reduced number of five stressful life events. 

Change of address in the first trimester was common in cases (41%) and was associated with increased risk of gastroschisis independently of all other risk factors studied. Change of address would be expected to correlate with social problems and relationship difficulties [21] as well as poverty but in our data the increased risk was independent of these variables. Previous studies of residential mobility in pregnancy and congenital malformations as reviewed by Bell and Belanger [40] have focussed on residence as a proxy for environmental exposures and the introduction of exposure misclassification by residential moves. However, change of residence may lead to other adverse factors such as disruption of antenatal care and loss of social support, and as we have postulated, to increased stress. Stress pathways need to be considered as confounding factors in studies of environmental exposures. However, in our study we could not rule out the possibility that residential moves were related to environmental exposures rather than increased maternal stress. 

### Conclusions

We report for the first time that the risk of gastroschisis is strongly associated with stressful life events including moving home in the first trimester. Though maternal stress was associated with increased rates of adverse lifestyle factors such as smoking and poor diet maternal stress was independently associated with increased risk at a level equivalent to the independent effect of maternal smoking. 

## Supporting Information

Table S1 **Gastroschisis case and control sample lifestyle, socio-demographic and nutrition characteristics by individual major stressful life events (Serious relationship difficulties, Legal or financial problems, Victim of violence or crime, Serious illness or injury, Death of someone close).**
(DOCX)
