# Supplementary material for: The Role of Maternal Stress in Early Pregnancy in the Aetiology of Gastroschisis: An Incident Case Control Study
Source: PLoS One. 2013 Nov 8;8(11):e80103. doi: 10.1371/journal.pone.0080103 (PMC3832654; doi:10.1371/journal.pone.0080103)
Supplement: Table S1 — Gastroschisis case and control sample lifestyle, socio-demographic and nutrition characteristics by individual major stressful life events (Serious relationship difficulties, Legal or financial problems, Victim of violence or crime, Serious illness or injury, Death of someone close). (DOCX) [file pone.0080103.s001.docx]

**Table S2a – Lifestyle, socio-demographic and nutrition characteristics by individual major stressful life events**

|  | | **Serious relationship difficulties for you / partner in first trimester** | | | | | | | |  | **Legal or financial problems for you / partner in first trimester** | | | | | | | |  | **Victim of violence or crime for you / someone close in first trimester** | | | | | | | |
| --- | --- | --- | --- | --- | --- | --- | --- | --- | --- | --- | --- | --- | --- | --- | --- | --- | --- | --- | --- | --- | --- | --- | --- | --- | --- | --- | --- |
|  |  | **No** | | | | **Yes** | | | |  | **No** | | | | **Yes** | | | |  | **No** | | | | **Yes** | | | |
|  |  | **Control** | | **Case** | | **Control** | | **Case** | |  | **Control** | | **Case** | | **Control** | | **Case** | |  | **Control** | | **Case** | | **Control** | | **Case** | |
|  |  | **n** | **(%)** | **n** | **(%)** | **n** | **(%)** | **n** | **(%)** |  | **n** | **(%)** | **n** | **(%)** | **n** | **(%)** | **n** | **(%)** |  | **n** | **(%)** | **n** | **(%)** | **n** | **(%)** | **n** | **(%)** |
| Cigarettes smoked per day in first trimester | 0 | 99 | (57.9) | 17 | (30.4) | 19 | (41.3) | 9 | (25.7) |  | 105 | (55.9) | 21 | (30.0) | 13 | (44.8) | 5 | (23.8) |  | 109 | (53.4) | 21 | (29.2) | 9 | (69.2) | 5 | (26.3) |
|  | >0-10 | 50 | (29.2) | 26 | (46.4) | 21 | (45.7) | 16 | (45.7) |  | 60 | (31.9) | 30 | (42.9) | 11 | (37.9) | 12 | (57.1) |  | 69 | (33.8) | 32 | (44.4) | 2 | (15.4) | 10 | (52.6) |
|  | >10 | 22 | (12.9) | 13 | (23.2) | 6 | (13.0) | 10 | (28.6) |  | 23 | (12.2) | 19 | (27.1) | 5 | (17.2) | 4 | (19.0) |  | 26 | (12.7) | 19 | (26.4) | 2 | (15.4) | 4 | (21.1) |
|  |  |  |  |  |  |  |  |  |  |  |  |  |  |  |  |  |  |  |  |  |  |  |  |  |  |  |  |
| Binge drinker (≥6 units in one sitting/day) in first trimester | No | 103 | (60.9) | 23 | (41.8) | 31 | (68.9) | 17 | (48.6) |  | 118 | (63.8) | 31 | (44.9) | 16 | (55.2) | 9 | (42.9) |  | 127 | (62.9) | 32 | (44.4) | 7 | (58.3) | 8 | (44.4) |
|  | Yes | 66 | (39.1) | 32 | (58.2) | 14 | (31.1) | 18 | (51.4) |  | 67 | (36.2) | 38 | (55.1) | 13 | (44.8) | 12 | (57.1) |  | 75 | (37.1) | 40 | (55.6) | 5 | (41.7) | 10 | (55.6) |
|  |  |  |  |  |  |  |  |  |  |  |  |  |  |  |  |  |  |  |  |  |  |  |  |  |  |  |  |
| Maternal age at childbirth | < 20 | 41 | (24.0) | 14 | (25.0) | 14 | (30.4) | 12 | (34.3) |  | 55 | (29.3) | 22 | (31.4) | 0 | (0.0) | 4 | (19.0) |  | 50 | (24.5) | 22 | (30.6) | 5 | (38.5) | 4 | (21.1) |
|  | 20 - 24.99 | 72 | (42.1) | 25 | (44.6) | 25 | (54.3) | 18 | (51.4) |  | 75 | (39.9) | 30 | (42.9) | 22 | (75.9) | 13 | (61.9) |  | 90 | (44.1) | 32 | (44.4) | 7 | (53.8) | 11 | (57.9) |
|  | 25+ | 58 | (33.9) | 17 | (30.4) | 7 | (15.2) | 5 | (14.3) |  | 58 | (30.9) | 18 | (25.7) | 7 | (24.1) | 4 | (19.0) |  | 64 | (31.4) | 18 | (25.0) | 1 | (7.7) | 4 | (21.1) |
|  |  |  |  |  |  |  |  |  |  |  |  |  |  |  |  |  |  |  |  |  |  |  |  |  |  |  |  |
| NS-SEC classification (of mother) | Managerial & professional / intermediate | 48 | (28.1) | 9 | (16.1) | 10 | (21.7) | 3 | (8.6) |  | 52 | (27.7) | 9 | (12.9) | 6 | (20.7) | 3 | (14.3) |  | 54 | (26.5) | 10 | (13.9) | 4 | (30.8) | 2 | (10.5) |
|  | Routine & manual | 53 | (31.0) | 28 | (50.0) | 13 | (28.3) | 12 | (34.3) |  | 58 | (30.9) | 33 | (47.1) | 8 | (27.6) | 7 | (33.3) |  | 64 | (31.4) | 32 | (44.4) | 2 | (15.4) | 8 | (42.1) |
|  | Unemployed | 60 | (35.1) | 16 | (28.6) | 16 | (34.8) | 17 | (48.6) |  | 61 | (32.4) | 23 | (32.9) | 15 | (51.7) | 10 | (47.6) |  | 71 | (34.8) | 24 | (33.3) | 5 | (38.5) | 9 | (47.4) |
|  | Student | 10 | (5.8) | 3 | (5.4) | 7 | (15.2) | 3 | (8.6) |  | 17 | (9.0) | 5 | (7.1) | 0 | (0.0) | 1 | (4.8) |  | 15 | (7.4) | 6 | (8.3) | 2 | (15.4) | 0 | (0.0) |
|  |  |  |  |  |  |  |  |  |  |  |  |  |  |  |  |  |  |  |  |  |  |  |  |  |  |  |  |
| Typical number of fruit or vegetables portions eaten per week (excluding potatoes) | 0 - 6 portions | 39 | (22.9) | 19 | (35.2) | 8 | (17.8) | 12 | (35.3) |  | 43 | (23.1) | 25 | (36.8) | 4 | (13.8) | 6 | (30.0) |  | 46 | (22.8) | 27 | (38.6) | 1 | (7.7) | 4 | (22.2) |
|  | 7 - 13 portions | 44 | (25.9) | 15 | (27.8) | 11 | (24.4) | 12 | (35.3) |  | 51 | (27.4) | 21 | (30.9) | 4 | (13.8) | 6 | (30.0) |  | 49 | (24.3) | 21 | (30.0) | 6 | (46.2) | 6 | (33.3) |
|  | 14 – 20 portions | 34 | (20.0) | 10 | (18.5) | 14 | (31.1) | 4 | (11.8) |  | 38 | (20.4) | 11 | (16.2) | 10 | (34.5) | 3 | (15.0) |  | 44 | (21.8) | 9 | (12.9) | 4 | (30.8) | 5 | (27.8) |
|  | 21+ portions | 53 | (31.2) | 10 | (18.5) | 12 | (26.7) | 6 | (17.6) |  | 54 | (29.0) | 11 | (16.2) | 11 | (37.9) | 5 | (25.0) |  | 63 | (31.2) | 13 | (18.6) | 2 | (15.4) | 3 | (16.7) |
|  |  |  |  |  |  |  |  |  |  |  |  |  |  |  |  |  |  |  |  |  |  |  |  |  |  |  |  |
| Duration in weeks of folic acid supplementation during first trimester | < 6 out of first 12 weeks | 48 | (28.1) | 26 | (46.4) | 15 | (32.6) | 20 | (57.1) |  | 54 | (28.7) | 34 | (48.6) | 9 | (31.0) | 12 | (57.1) |  | 57 | (27.9) | 36 | (50.0) | 6 | (46.2) | 10 | (52.6) |
|  | ≥ 6 out of first 12 weeks | 123 | (71.9) | 30 | (53.6) | 31 | (67.4) | 15 | (42.9) |  | 134 | (71.3) | 36 | (51.4) | 20 | (69.0) | 9 | (42.9) |  | 147 | (72.1) | 36 | (50.0) | 7 | (53.8) | 9 | (47.4) |
|  |  |  |  |  |  |  |  |  |  |  |  |  |  |  |  |  |  |  |  |  |  |  |  |  |  |  |  |
| Social support available (Emotional, Financial, Help with daily tasks) | No (lack of 1+ support) | 20 | (11.7) | 8 | (14.3) | 8 | (17.4) | 13 | (37.1) |  | 19 | (10.1) | 13 | (18.6) | 9 | (31.0) | 8 | (38.1) |  | 26 | (12.7) | 14 | (19.4) | 2 | (15.4) | 7 | (36.8) |
|  | Yes (all 3 supports) | 151 | (88.3) | 48 | (85.7) | 38 | (82.6) | 22 | (62.9) |  | 169 | (89.9) | 57 | (81.4) | 20 | (69.0) | 13 | (61.9) |  | 178 | (87.3) | 58 | (80.6) | 11 | (84.6) | 12 | (63.2) |
|  |  |  |  |  |  |  |  |  |  |  |  |  |  |  |  |  |  |  |  |  |  |  |  |  |  |  |  |

**Table S2a (continued) – Lifestyle, socio-demographic and nutrition characteristics by individual major stressful life events**

|  | | **Serious illness or injury for you / someone close in first trimester** | | | | | | | |  | **Death of someone close in first trimester** | | | | | | | |
| --- | --- | --- | --- | --- | --- | --- | --- | --- | --- | --- | --- | --- | --- | --- | --- | --- | --- | --- |
|  |  | **No** | | | | **Yes** | | | |  | **No** | | | | **Yes** | | | |
|  |  | **Control** | | **Case** | | **Control** | | **Case** | |  | **Control** | | **Case** | | **Control** | | **Case** | |
|  |  | **n** | **(%)** | **n** | **(%)** | **n** | **(%)** | **n** | **(%)** |  | **n** | **(%)** | **n** | **(%)** | **n** | **(%)** | **n** | **(%)** |
| Cigarettes smoked per day in first trimester | 0 | 96 | (51.9) | 17 | (22.4) | 22 | (68.8) | 9 | (60.0) |  | 108 | (53.5) | 20 | (24.7) | 10 | (66.7) | 6 | (60.0) |
|  | >0-10 | 62 | (33.5) | 38 | (50.0) | 9 | (28.1) | 4 | (26.7) |  | 66 | (32.7) | 39 | (48.1) | 5 | (33.3) | 3 | (30.0) |
|  | >10 | 27 | (14.6) | 21 | (27.6) | 1 | (3.1) | 2 | (13.3) |  | 28 | (13.9) | 22 | (27.2) | 0 | (0.0) | 1 | (10.0) |
|  |  |  |  |  |  |  |  |  |  |  |  |  |  |  |  |  |  |  |
| Binge drinker (≥6 units in one sitting/day) in first trimester | No | 110 | (60.1) | 36 | (48.0) | 24 | (77.4) | 4 | (26.7) |  | 122 | (61.3) | 37 | (46.3) | 12 | (80.0) | 3 | (30.0) |
|  | Yes | 73 | (39.9) | 39 | (52.0) | 7 | (22.6) | 11 | (73.3) |  | 77 | (38.7) | 43 | (53.8) | 3 | (20.0) | 7 | (70.0) |
|  |  |  |  |  |  |  |  |  |  |  |  |  |  |  |  |  |  |  |
| Maternal age at childbirth | < 20 | 51 | (27.6) | 24 | (31.6) | 4 | (12.5) | 2 | (13.3) |  | 53 | (26.2) | 26 | (32.1) | 2 | (13.3) | 0 | (0.0) |
|  | 20 - 24.99 | 80 | (43.2) | 34 | (44.7) | 17 | (53.1) | 9 | (60.0) |  | 88 | (43.6) | 34 | (42.0) | 9 | (60.0) | 9 | (90.0) |
|  | 25+ | 54 | (29.2) | 18 | (23.7) | 11 | (34.4) | 4 | (26.7) |  | 61 | (30.2) | 21 | (25.9) | 4 | (26.7) | 1 | (10.0) |
|  |  |  |  |  |  |  |  |  |  |  |  |  |  |  |  |  |  |  |
| NS-SEC classification  (of mother) | Managerial & professional / intermediate | 52 | (28.1) | 12 | (15.8) | 6 | (18.8) | 0 | (0.0) |  | 52 | (25.7) | 12 | (14.8) | 6 | (40.0) | 0 | (0.0) |
|  | Routine & manual | 51 | (27.6) | 32 | (42.1) | 15 | (46.9) | 8 | (53.3) |  | 65 | (32.2) | 36 | (44.4) | 1 | (6.7) | 4 | (40.0) |
|  | Unemployed | 66 | (35.7) | 26 | (34.2) | 10 | (31.3) | 7 | (46.7) |  | 68 | (33.7) | 27 | (33.3) | 8 | (53.3) | 6 | (60.0) |
|  | Student | 16 | (8.6) | 6 | (7.9) | 1 | (3.1) | 0 | (0.0) |  | 17 | (8.4) | 6 | (7.4) | 0 | (0.0) | 0 | (0.0) |
|  |  |  |  |  |  |  |  |  |  |  |  |  |  |  |  |  |  |  |
| Typical number of fruit or vegetables portions eaten per week (excluding potatoes) | 0 - 6 portions | 40 | (21.7) | 24 | (32.4) | 7 | (22.6) | 7 | (50.0) |  | 45 | (22.5) | 27 | (34.6) | 2 | (13.3) | 4 | (40.0) |
|  | 7 - 13 portions | 48 | (26.1) | 26 | (35.1) | 7 | (22.6) | 1 | (7.1) |  | 50 | (25.0) | 26 | (33.3) | 5 | (33.3) | 1 | (10.0) |
|  | 14 - 20 portions | 41 | (22.3) | 12 | (16.2) | 7 | (22.6) | 2 | (14.3) |  | 42 | (21.0) | 12 | (15.4) | 6 | (40.0) | 2 | (20.0) |
|  | 21+ portions | 55 | (29.9) | 12 | (16.2) | 10 | (32.3) | 4 | (28.6) |  | 63 | (31.5) | 13 | (16.7) | 2 | (13.3) | 3 | (30.0) |
|  |  |  |  |  |  |  |  |  |  |  |  |  |  |  |  |  |  |  |
| Duration in weeks of folic acid supplementation during first trimester | < 6 out of first 12 weeks | 56 | (30.3) | 37 | (48.7) | 7 | (21.9) | 9 | (60.0) |  | 59 | (29.2) | 39 | (48.1) | 4 | (26.7) | 7 | (70.0) |
|  | ≥ 6 out of first 12 weeks | 129 | (69.7) | 39 | (51.3) | 25 | (78.1) | 6 | (40.0) |  | 143 | (70.8) | 42 | (51.9) | 11 | (73.3) | 3 | (30.0) |
|  |  |  |  |  |  |  |  |  |  |  |  |  |  |  |  |  |  |  |
| Social support available (Emotional, Financial, Help  with daily tasks) | No (lack of 1+ support) | 26 | (14.1) | 15 | (19.7) | 2 | (6.3) | 6 | (40.0) |  | 26 | (12.9) | 17 | (21.0) | 2 | (13.3) | 4 | (40.0) |
|  | Yes (all 3 supports) | 159 | (85.9) | 61 | (80.3) | 30 | (93.8) | 9 | (60.0) |  | 176 | (87.1) | 64 | (79.0) | 13 | (86.7) | 6 | (60.0) |
|  |  |  |  |  |  |  |  |  |  |  |  |  |  |  |  |  |  |  |
